# Hospitalization for Short-Term Diabetes-Related Complications: Focus on Patients Aged over 85 Years

**DOI:** 10.3390/healthcare9040460

**Published:** 2021-04-14

**Authors:** Giuseppe Di Martino, Pamela Di Giovanni, Fabrizio Cedrone, Francesca Meo, Piera Scampoli, Ferdinando Romano, Tommaso Staniscia

**Affiliations:** 1 Department of Medicine and Ageing Sciences, “G. d’Annunzio” University of Chieti-Pescara, 66100 Chieti, Italy; tommaso.staniscia@unich.it; 2 Department of Pharmacy, “G. d’Annunzio” University of Chieti-Pescara, 66100 Chieti, Italy; pamela.digiovanni@unich.it; 3 School of Hygiene and Preventive Medicine, “G. d’Annunzio” University of Chieti-Pescara, 66100 Chieti, Italy; cedronefab@gmail.com (F.C.); Francesca.meo10@gmail.com (F.M.); piera.scampoli@gmail.com (P.S.); 4 Department of Infectious Diseases and Public Health, “La Sapienza” University of Rome, 00185 Rome, Italy; ferdinando.romano@uniroma1.it

**Keywords:** short-term diabetes complications, diabetes, elderly, hospitalization, epidemiology

## Abstract

(1) *Background*: The prevalence of diabetes in elderly people is frequently high. When occurring in the elderly, diabetes is often accompanied by complications and comorbidities, at least one in 60% and four or more in 40% of older people with diabetes. As far as short-term complications among the elderly are concerned, hypoglycemia and hyperglycemic crises prove to be frequent. The aim of this study was to investigate the difference in hospitalization for short-term diabetes complications in patients below and over 85 years of age. (2) *Methods*: Data were collected from hospital discharge records (HDRs) of all hospital admissions that occurred in Abruzzo Region, Italy, from 2006 to 2015. Only diabetic patients aged over 65 years were included. Outcomes included were diabetic ketoacidosis, hyperosmolar coma, hypoglycemic shock, iatrogenic hypoglycemic coma, and other diabetic comas. (3) *Results*: During the study period, 144,376 admissions were collected, 116,305 (80.56%) of which referred to patients below 85 years. Those aged over 85 years were significantly associated to all short-term diabetes-related complications with the exception of ketoacidosis. (4) *Conclusions*: In older diabetic patients, the avoidance of short-term diabetes complications are a greater concern than in younger patients. Diabetes management among very elderly patients should be tailored accordingly to patient characteristics.

## 1. Introduction

Diabetes mellitus is a worldwide epidemic with a prevalence that has been rapidly increasing. The prevalence of diabetes in elderly people is frequently high, though differences are reported among different countries. In the USA, more than 25% of older adults (aged > 65 years) are diagnosed with diabetes, and the incidence of diabetes diagnosis among patients aged over 65 years is 11.5 cases per 1000 persons per year [1,2]. In Europe, prevalence data are lower, showing an average 20% prevalence with some differences by nation [3]. In Italy, the prevalence of diabetes has reached 15–26%, with the highest percentage of diabetes cases (66.3%) occurring among patients aged over 65 years [4]. Diabetes is associated with macro- and microvascular complications, including cardiovascular (CV) disease, nephropathy, retinopathy, and neuropathy, but these typically occur as a result of the disease/complication after several years [5]. When occurring in the elderly, diabetes is frequently accompanied by complications and comorbidities, at least one in 60% and four or more in 40% of older people with diabetes [6]. Older adults are at high risk for the development of type 2 diabetes (T2D) due to the combined effects of increasing insulin resistance and impaired pancreatic islet function with aging. Age-related insulin resistance seems to be primarily associated with adiposity, sarcopenia, and physical inactivity [7]. Older adults with diabetes have the highest rates of major lower extremity amputation [8,9], myocardial infarction, visual impairment, and end-stage renal disease. Those aged 75 years and older show higher rates than those aged 65–74 years for most complications [10]. Recent trials have reported that intensive glycemic control can reduce complications, even though approximately eight years are needed before intensive glycemic control leads to a reduction in microvascular complications [11,12,13]. As far as short-term complications among the elderly are concerned, hypoglycemia and hyperglycemic crises prove to be frequent. Deaths from hyperglycemic crises are significantly higher in older adults, and, particularly, patients over 75 years of age report a double rate of emergency department admission for hypoglycemia than the general diabetic population [10,14]. In addition, intensive glycemic control is often also associated with increased rates of hypoglycemia, leading to serious consequences such as falls and hip fractures [12,13]. Despite a growing life expectancy, a few studies have investigated short-term complications among patients aged over 85 years. Therefore, the aim of this study was to investigate the difference in hospitalization for short-term diabetes-related complications (STDRCs) in patients below and over 85 years of age.

## 2. Materials and Methods

This retrospective study was conducted in Abruzzo, a region of Southern Italy, with approximately 1.3 million inhabitants and high prevalence of diabetes (7.4% of entire population) [4]. Data were collected from hospital discharge records (HDRs) of all hospital admissions that occurred in Abruzzo from 1 January 2006 to 31 December 2015. The HDRs collected information on admission source and type, admission and discharge dates, patient baseline demographics (age, gender, citizenship, birthplace, and residence), a principal and up to five additional diagnoses, a main procedure and up to five further procedures. Diagnoses and procedures were coded according to the International Classification of Disease, 9th Revision, Clinical Modification (ICD-9-CM, National Center for Health Statistics (NCHS) and the Centers for Medicare and Medicaid Services External, Atlanta, GE, USA). Only diabetic patients aged over 65 years were included in this study. Among patients’ comorbidities, all diagnosis referred to the Charlson Comorbidity Index (CCI) were extracted. The STDRCs’ main outcomes were diabetic ketoacidosis (code 250.1), hyperosmolar coma (code 250.2), hypoglycemic shock (code 250.8), iatrogenic hypoglycemic coma (code 251.0), and other diabetic coma (code 250.3).

### Statistical Analysis

Continuous variables were reported as the mean and standard deviation (SD). Categorical variables were reported as frequency and percentage. Pearson’s Chi-square test and Student’s *t*-test were performed to compare baseline variables between study groups, as appropriate. Multivariable logistic regression models adjusted for gender and comorbidities included in the CCI were performed to evaluate the association between STDRCs and age over 85 years. Statistical significance was set at *p*-value < 0.05. All analyses were performed with Stata^®^ version 15 (StataCorp LLC, College Station, TX, USA).

## 3. Results

### Patients Characteristics

During the study period, 144,376 admissions of diabetic patients aged over 65 years were collected, 116,305 (80.56%) of which referred to patients below 85 years of age. Diabetic patients aged over 84 years were more frequently female (17,948; 63.9%) and had different baseline characteristics compared with patients aged below 85 years as shown in Table 1.

In particular, patients over 85 years of age suffered more frequently of heart failure (26.5% versus 15.9%), cerebrovascular disease (20.9% versus 16.8%), COPD (14.7% versus 12.5%), dementia (5.4% versus 1.6%), and kidney disease (14.1% versus 9.8%). Conversely, patients aged below 85 were more frequently affected by cancer (7.9% versus 5.9%), ischemic heart disease (6.9% versus 5.5%), peripheral vascular disease (7.3% versus 4.9%), uncontrolled diabetes (16.1% versus 14.7%), and mild liver disease (5.5% versus 2.7%). During the study period, 3607 admissions (2.5%) were referred to STDRCs. The most frequent STDRC observed was hypoglycemic shock, occurring 1898 times (1.6%) among patients below 85 years and 523 times (1.9%) among patients over 85 years. As described in Table 2, logistic regression analysis showed that age over 85 years was significantly associated to all STDRCs with the exception of ketoacidosis.

## 4. Discussion

This study shows patients aged over 85 years suffer from different comorbidities with respect to patients aged below 85 years. In addition, age over 85 years was significantly associated to STDRCs [14]. Gender differences can be explained by the higher life expectancy of women. In addition, as previously reported in epidemiological reports [15,16], hospitalized women were older than men, but male sex was more affected than women with regards to cumulative illness burden with higher short- and long-term mortality following hospitalization [16]. Moreover, it is known that elderly diabetic individuals have different characteristics if compared with younger diabetic individuals, highlighting the need for a personalized treatment for older diabetics. Older subjects are actually very heterogeneous in terms of functional status, comorbidities, and degree of frailty [2]. Living conditions also play a role in the management of a diabetic patient, as the subject may either be living independently or in a supporting facility. Moreover, given the frequent exclusion from randomized controlled trials, further investigation is needed for developing supporting treatment strategies for patients over 85 years of age [17]. In addition, older diabetic adults frequently need polypharmacy, increasing the risk of drug adverse events and potentially life-threatening interactions [18]. One of the main treatment objectives for older diabetic patients is glycemic control in order to avoid micro- and macrovascular complications [19]. Nevertheless, intensive glucose control can lead to hypoglycemia, frequently occurring during intensive anti-hyperglycemic treatment conducted with insulin or sulphonylureas/glinides. Hypoglycemia causes an increase in incident falls and the exacerbation of pre-existing comorbidities [20,21]. The high risk of this complication in diabetes care was highlighted by the HYPOTHESIS study conducted in 46 Italian centers, revealing that considerably high hospitalization and deathrates occur when severe hypoglycemic events require referral to the emergency department, especially in elderly and frail patients [22]. Recently, the multicenter retrospective HYPOS-1 study investigated further the incidence and risk factors of hypoglycemia in more than 2000 Italian patients with diabetes, highlighting the heavy economic burden of hypoglycemic hospitalization [23,24,25,26]. In addition, the results of a retrospective observational study from USA evaluated the impact of hypoglycemic events on acute CV events over a 2 year period, showing how patients experiencing hypoglycemic events had a significantly higher risk of acute CV events [27]. Among hospitalized patients, with diabetes, hypoglycemia is associated with increased length of hospitalization and in-hospital mortality, as reported in a previous study [28]. It is also important to consider the effect of repeated episodes of iatrogenic hypoglycemia, which attenuates the autonomic response to subsequent hypoglycemia. This process resets the glycemic threshold to a lower glucose level and reduces the subject’s ability to perceive the onset of hypoglycemic symptoms. Clinical experience indicates that many individuals with longstanding insulin-treated diabetes have impaired hypoglycemia awareness [29]. Hypoglycemia and, above all, impaired hypoglycemia awareness, have been linked to cognitive dysfunction [30]. In addition, a decline in intellectual capacity has been noted with progressive loss of hypoglycemia awareness [31]. Furthermore, hyperglycemic hyperosmolar coma (HHC) is a hyperglycemic complication typical of older patients, in comparison with diabetic ketoacidosis (DKA) that mostly occurs among younger subjects. Hyperglycemic hyperosmolar coma is most frequently reported in older patients with T2D with an intercurrent illness such as infection, surgery or ischemic events, and is associated with a higher mortality rate than DKA [30,31,32]. Poor adherence to medical therapy and new diabetes onset are a less common precipitating cause of HHC than DKA. Differently, age is one of the leading factors associated with HHC compared with DKA [33]. In addition, HHC is associated with a higher mortality rate among elderly diabetic patients [34]. The patient-centered approach advocates that several patient-specific characteristics should be taken into account when determining glycemic targets in older adults. The patient-specific characteristics include psycho-socioeconomic considerations, risk for hypoglycemia, patient age, duration of diabetes, and other comorbid conditions [5].

### Strengths and Limitations

The strength of this study is the large sample analyzed and the long study period considered. However, the results of this study should be considered in the light of the following limitations. First, the identification of diagnosis was based on ICD-9-CM codes that did not take into account the severity of conditions. Second, the use of administrative data may be limited by the reliability of certain types of information such as drugs therapy and clinical information. Third, all diagnoses codes could not be reported due to the lack of information or to miscoding.

## 5. Conclusions

Older adults with diabetes are a heterogeneous group with several comorbidities and functional disabilities. The overall goals of diabetes management in older adults are similar to those in younger adults and include the management of both hyperglycemia and risk factors. Nevertheless, metabolism in the elderly is distinctly different, and the approach to therapy needs to be tailored accordingly. In frail older patients with diabetes, the avoidance of STDRCs and drug interactions are a much greater concern than in younger patients with diabetes.

## Figures and Tables

**Table 1:** Comparison of characteristics between patients over and below 85 years of age.

*N* (%)	<85 Years (*n* = 116,305)	≥85 Years (*n* = 28,071)	*p*-Value
Male gender	63,282 (54.4)	10,123 (36.6)	<0.001
Type 1 diabetes	13,526 (11.6)	2714 (9.7)	<0.001
Heart failure	18,506 (15.9)	7429 (26.5)	<0.001
Ischemic heart disease	8014 (6.9)	1538 (5.5)	<0.001
Peripheral vascular disease	8432 (7.3)	1388 (4.9)	<0.001
Stroke	19,570 (16.8)	5877 (20.9)	<0.001
Dementia	1846 (1.6)	1526 (5.4)	<0.001
COPD	14,570 (12.5)	4117 (14.7)	<0.001
Rheumatologic disease	1085 (0.9)	208 (0.7)	0.002
Peptic ulcer	713 (0.6)	181 (0.6)	0.543
Uncontrolled diabetes	18,774 (16.1)	4117 (14.7)	<0.001
Emi/paraplegia	672 (0.6)	157 (0.6)	0.713
Cancer	9203 (7.9)	1647 (5.9)	<0.001
Metastasis	2450 (2.1)	358 (1.3)	<0.001
Mild hepatic disease	6413 (5.5)	747 (2.7)	<0.001
Severe hepatic disease	1220 (1.1)	104 (0.4)	<0.001
HIV	68 (0.1)	2 (0.0)	<0.001
Renal disease	11,405 (9.8)	3972 (14.1)	<0.001

COPD: Chronic obstructive pulmonary disease.

**Table 2:** Logistic regression analysis showing the association between age over 85 years and short-term diabetes-related complication (STDRC).

Diagnosis	<85 Years *n* (%)	≥85 Years *n* (%)	OR(95%CI)	*p*-Value
Ketoacidosis	1134 (1.0)	296 (1.1)	1.11 (0.97–1.27)	0.155
Hyperosmolar state/coma	889 (0.8)	302 (1.1)	1.40 (1.22–1.61)	<0.001
Iatrogenic hypoglycemic coma	150 (0.13)	68 (0.24)	1.16 (1.05–1.28)	<0.001
Hypoglycemic shock	1898 (1.6)	523 (1.9)	1.82 (1.35–2.45)	0.004
Others diabetic coma	570 (0.5)	198 (0.7)	1.41 (1.19–1.66)	<0.001
